# *Información es poder* (information is power): menopause knowledge, attitudes, and experiences in midlife Hispanic women and Latinas

**DOI:** 10.1186/s12905-024-03434-z

**Published:** 2024-12-02

**Authors:** Yamnia I. Cortés, Andrea Cazales, Valentina Mărginean, Mayra Duran, Lorena Trocel

**Affiliations:** 1 College of Nursing, University of Iowa, 50 Newton Road, Iowa City, IA 52242 USA; 2 School of Nursing, University of North Carolina at Chapel Hill, Carrington Hall, Chapel Hill, NC 27599 USA; 3 Charles R. Drew University of Medicine and Science, 1731 E 120th St, Los Angeles, CA 90059 USA

**Keywords:** Hispanic/Latinas, Culturally-tailored intervention, Health disparities, Menopause knowledge, Menopause attitudes

## Abstract

**Background:**

Latinas constitute nearly 20% of midlife women in the United States (U.S.), but remain underrepresented in menopause research. Many midlife Latinas are disadvantaged by limited English proficiency, less formal education, living below the federal poverty level, lack of health insurance, and social isolation and discrimination — factors that negatively affect menopause-related symptoms and health outcomes. This study aimed to understand knowledge, attitudes, and experiences of the menopause transition among midlife Latinas.

**Methods:**

We conducted a qualitative descriptive study using five focus groups with 29 Hispanic/Latina midlife women. An emergent content analysis was performed by four bilingual coders. Data on socio-demographics, menopausal symptoms, menopause knowledge, and attitudes toward menopause and hormone therapy were collected over the phone. Descriptive statistics were performed to characterize study participants.

**Results:**

Participants were aged 50.3 ± 6.3 years, 45% postmenopausal, 79% viewed menopause positively, and 55% reported having “little knowledge” about menopause. Seven themes emerged: 1) menopause is a stage of life (*una etapa de vida*); 2) not wanting to become an old lady (*no quererme hacer viejita)*; 3) in our culture, we do not ask [about menopause]; 4) family dynamics; 5) each body is different (*todo cuerpo es diferente)*; 6) menopause self-management and treatment options; 7) information is power *(información es poder)*.

**Conclusions:**

Although Latinas reported having a positive view of menopause, we found a need for culturally-tailored comprehensive menopause education. The importance of involving family members in menopause education was also revealed. Our next steps are to determine the best menopause messaging strategies and educational formats for midlife Latinas.

**Supplementary Information:**

The online version contains supplementary material available at 10.1186/s12905-024-03434-z.

## Background

In 2019, 20% of women aged 45–54 years in the United States identified as Hispanic or Latino, regardless of their racial identity [1]. Latinas are a heterogeneous group with varied sociocultural and historical experiences that may impact their menopause transition (MT). Nearly 30% of Latinas have limited English proficiency, 18% live below the federal poverty level, and 24% lack health insurance [2]—factors related to more frequent and/or severe menopause symptoms [3]. Compared to non-Latina White women, Latinas experience more vasomotor symptoms, depressive symptoms, sleep disturbances, and have a more adverse cardiometabolic risk profile during the MT [4–6]. While several epidemiologic investigations have enhanced our understanding of the MT, to date, menopause research has mostly focused on non-Latina White women. Consequently, there is a dearth of information on the knowledge, attitudes, and experiences of Latinas during the MT.

The experience of menopause is characterized by cultural values, personal challenges, and roles within the family and society [4]. Additionally, how women perceive and cope with the natural changes that occur during the MT is influenced by multiple sociocultural factors such as acculturation, discrimination, and social support [3, 7, 8]. Negative attitudes towards menopause have been related to more frequent reports of symptoms during the MT. [9] Although research suggests Latinas have a positive attitude toward menopause [10], midlife Latinas report greater anxiety and over 60% feel unprepared for the MT [11]. Greater menopause knowledge has been related to better symptom management [12]. However, there is a lack of comprehensive culturally-tailored education on menopause for Latinas, and few resources in Spanish, the second most common language in the U.S [13].Additionally, there have been limited opportunities for community participation in Latina-led research endeavors due to educational barriers and institutional mistrust which potentially limits engagement with minoritized groups [13]. The overall purpose of this study was to better understand the experience of the MT among midlife Latinas. Findings from this study will contribute to the development of menopause education tools for Latinas, which may improve menopause-related outcomes and enhance quality of life.

## Methods

### Design

We conducted a qualitative descriptive study, which enables researchers to investigate, describe, and summarize a phenomenon in everyday terms [14]. We employed an interpretive constructivist research paradigm to explore how participants perceive the MT. An interpretive constructivist paradigm emphasizes describing encounters, processes, or cultures from the perspective of participants [15]. This study was conducted in accordance with the Declaration of Helsinki, and approval was granted by The University of North Carolina at Chapel Hill (UNC) Institutional Review Board.

### Participants and setting

We recruited participants using flyers at the University of North Carolina at Chapel Hill, community organizations providing services to Latinas, and Latino-owned businesses in the surrounding area. Moreover, we recruited women from a contact list of individuals who were ineligible for a concurrent pilot study to reduce cardiovascular disease risk in perimenopausal Latinas (K23MD014767; PI: Cortés) and expressed interest in participating in future menopause-related studies. A research assistant screened participants over the phone to confirm eligibility. Eligible participants were women aged 35–60 years who self-identified as Hispanic/Latina. Because of our interest in menopause attitudes and knowledge, we did not exclude premenopausal women. Focus groups were conducted online in a private setting via a videoconferencing platform.

### Data collection

Before each focus group session, a research assistant contacted participants by phone and completed a menopause health questionnaire (lasting approximately 15 min) to collect socio-demographic information (i.e., age, education, employment), current medications, and comorbid medical conditions (Supplementary 1). The questionnaire was purchased and adapted from The Menopause Society health questionnaire (https://menopause.org/professional-resources), and translated into Spanish by two bilingual team members. Participants categorized their reproductive cycles in accordance to common nomenclature in both clinical research and health promotion [16, 17]. They categorized themselves as premenopausal if they were having regular menstrual cycles, perimenopausal if they had irregular or unpredictable cycles (persistent ≥ 7-day difference in length of consecutive cycles), and postmenopausal if it had been ≥ 12 months since the last menstrual period. Participants were asked how bothered they were by individual menopause symptoms in the past two weeks (not bothered at all; a little bit; quite a bit; extremely). Finally, participants were asked whether they had positive or negative views on menopause and hormone therapy.

We conducted five focus groups in Spanish with 5–7 participants in each. Two bilingual (English, Spanish) research assistants who understand the participants’ sociocultural context led the focus groups using a semi-structured interview guide developed in Spanish and English for this study. We have included the English version in Supplementary 2. The Principal Investigator, who is also a bilingual Latina (English, Spanish), served as a note-taker and assisted with any technical issues (i.e., participants logging in and anonymizing themselves). Since all participants spoke and understood Spanish, focus groups were conducted in Spanish. All sessions, lasting approximately two hours, were audio recorded and transcribed verbatim for qualitative analysis. At the end of each focus group, the research team presented a 30-min menopause education module.

### Data analysis

Coding was completed in Spanish by four bilingual coders. Coders independently read the focus group transcripts in an iterative manner and met weekly to discuss their initial impressions and to develop the coding scheme. The analysis was completed manually in Microsoft Word and organized using a Microsoft Excel spreadsheet. In the first cycle of coding, the analysts used an In Vivo Coding strategy to create codes from the participant’s own words or short phrases [18]. Coders met weekly to revise the codes and their definitions based on which best fit the data. Once the coders agreed on the final list of codes and definitions, they individually applied the codes to the transcripts and assessed intercoder agreement (# of agreement/# of coding decisions) in Microsoft Excel at > 90% agreement. Finally, the coders met until they reached a consensus on how to group the data into categories and themes based on the patterns that emerged from the data. As in previous qualitative studies conducted in Spanish [19, 20], the emergent themes and respective quotes were translated from Spanish to English during the writing of this report by the bilingual research team familiar with the participants’ sociocultural context.

## Results

Focus group participants were on average aged 50.3 ± 6.3 years, 64% identified as Mexican, 71% were employed, and 96% were uninsured (Table 1). Half of the participants were postmenopausal, 43% perimenopausal, and 7% premenopausal. The symptoms most frequently reported as “quite a bit”/”extremely” bothersome were joint pain (39%), sleep disturbances (39%), fatigue (29%), vasomotor symptoms (25%), and weight gain/bloated (25%). Eighty-two percent of women viewed menopause positively and 61% viewed hormone therapy negatively (“I do not support the use of menopause hormone therapy”). Nearly 60% of focus group participants reported having “little knowledge” about menopause.

**Table 1:** Focus group participants characteristics (*n* = 28)*

Characteristics	Mean ± SD or N (%)
Age (years)	50.3 ± 6.3
Latina background Mexican Central American (i.e., Honduran, Guatemalan) South American (i.e., Colombia, Venezuela) Caribbean (i.e., Dominican, Puerto Rican)	18 (64.3) 4 (14.2) 5 (17.9) 1 (3.6)
Employed	20 (71.4)
No health insurance coverage	27 (96.4)
Menopausal status Premenopausal Perimenopausal Postmenopausal	2 (7.1) 12 (42.9) 15 (50.0)
Top 5 symptoms reported as quite a bit/extremely bothersome: Joint pain Sleep disturbances Fatigue Vasomotor symptoms Weight gain/bloated	11 (39.3) 11 (39.3) 8 (28.6) 7 (25.0) 7 (25.0)
How do you view menopause? Positive (e.g., no more worry about contraception, new phase) Negative (e.g., loss of fertility, loss of youth) Other	23 (82.1) 3 (10.7) 2 (7.1)
What are your views on hormone therapy for menopause? Positive (e.g., hormone therapy is appropriate for some women) Negative (e.g., I don’t support the use of hormone therapy) Other	9 (32.1) 17 (60.7) 2 (7.1)
How would you rate your knowledge about menopause? Very good Good/Fair Little knowledge	2 (7.1) 10 (35.7) 16 (57.1)

^*^Note. One participant did not complete survey

Seven themes emerged from the data (Table 2): 1) menopause is a stage of life (*una etapa de vida*); 2) not wanting to become an old lady (*no quererme hacer viejita)*; 3) in our culture, we do not ask questions [about menopause]; 4) family dynamics; 5) each body is different (*todo cuerpo es diferente)*; 6) menopause self-management and treatment options; 7) information is power *(información es poder)*.

**Table 2:** Final coding scheme and themes

Code	Definition/Meaning	Themes
Pérdida del período (*Loss of period*)	Describe la menopausia formalmente, no tener período, no tener menstruación (*Describes menopause formally, no period, no menstruation.)*	**Una etapa de la vida** **(*A stage of life)***
Una etapa de la vida normal (*A normal life stage)*	Describe la menopausia como una etapa normal. También refiere a la percepción de familiares, amistades, o proveedor de salud de la menopausia es algo normal. (*Describes menopause as a normal stage. Refers to the perception of family, friends, or healthcare providers that menopause is normal*)	
Experencia positiva de menopausia (*Positive experience of menopause*)	Participantes hablan de las cosas positivas de la menopausia, o de los resultados positivos después de la menopausia. *(Participants discuss the positive things about menopause, or positive outcomes after menopause)*	
Experencia negativa de menopausia (*Negative experience of menopause*)	Se refiere a la menopausia como algo negativo. Difiere de la enumeración de los síntomas de la menopausia. *(Refer to menopause as a negative experience. It differs from listing symptoms.)*	
Ya no somos jóvenes (*We are no longer young*)	Cuando la participante describe una conciencia, percepción, o sentimiento de ella misma como vieja. También cuando otras personas las perciben o las hacen sentir vieja. (*When the participant describes an awareness, perception, or feeling of herself as old. Also, when others view them or make them feel old)*	**No querer hacerme viejita** (N*ot wanting to become an old lady)*
Gente callada, reservada, no pregunta (*Quiet, reserved people, do not ask questions*)	Las personas en la cultura latina no conversan de la menopausia, es diferente en Estados Unidos. (*People in the Latino culture do not talk about menopause, it is different in the United States.*)	**En nuestra cultura no hacemos preguntas** (*In our culture, we do not ask questions [about menopause]*)
No tengo confianza preguntar (*I am not confident to ask*)	Cuando no se sienten cómodas para conversar y hacer preguntas sobre la menopausia. (*When they do not feel comfortable to discuss and ask questions about the menopause.*)	
Personas con las que vive deben saber mas (*People you live with should know more*)	Participante quisiera que sus familiares u otras personas en su hogar sepan sobre la menopausia. (*Participant would like family members or other people in your household to know about menopause.*)	**Family dynamics**
Familia sientan mas empatia (*Family feel more empathy*)	Refiere a la necesidad de la familia saber sobre la menopausia para tener más simpatía y soporte. (*Refers to the need of the family to know about menopause to have more sympathy and support*.)	
No lo entienden *(They do not understand)*	Los familiares no entienden, aunque saben que la persona pasa por la menopausia. (*Family members do not understand, even though they know the person is going through menopause.)*	
Me entienden (familia) *(They understand me (My family))*	Este code se usa cuando las participantes describen el soporte que reciben de la familia. (*This code is used when participants describe the support they receive from the family.)*	
Estas loca (*She is crazy*)	Este code se usa cuando las participantes describen lo que su esposo/familia piensa de ella (loca, mal de la cabeza) durante la perimenopausia. (*This code is used when the participants describe what their husband/family thinks of them (crazy, sick in the head) during perimenopause*.)	
Los que viven a mi alrededor sufren mucho (*Those who live around me suffer a lot*)	El código hace referencia al modo en que la menopausia ha afectado negativamente a las familias: las participantes se sienten culpables o como una carga. (*Code refers to the way menopause has negatively affected families—participants feel guilty, or like a burden.)*	
Primeros cambios corporals (*First body changes*)	Cambios o síntomas iniciales y no está conciente que esos cambios están asociados con la menopausia. (*Initial changes or symptoms and is unaware that those changes are associated with menopause.*)	**Todo cuerpo es diferente** **(*each body is different*)**
Problemas de salud que no relaciono con la menopausia (*Health problems I do not associate with menopause*)	Describe un historial o síntomas que la participante no atribuye a la menopausia. (*Describes a history or symptoms that the participant does not attribute to menopause.*)	
Apetito sexual (*Sexual appetite*)	Síntomas que experimentan de la menopausia (*Menopause symptoms they experience)*	
Sed (*Thirst*)		
Hinchada (*Swollen*)		
Aumentar peso (*Weight gain*)		
Cansada (*Tired)*		
Calores (*Hot flashes)*		
Artritis (*Arthitis)*		
Alergias (*Allergies)*		
Caída del Cabello (*Hair loss*)		
Migraña (*Migraines*)		
Cambio de humor/genio (*Mood changes*)		
Insomnia/dificultad de dormir (*Insomnia/difficulty sleeping*)		
Todo cuerpo es diferente (*Each body is different*)	Cuando las participantes comparan y notan que los síntomas o la experencia de cada persona es diferente (*When participants compare and note that each person's symptoms or experience is different.*)	
No senti cambios (*I felt no change*)	El código se refiere a las participantes que afirman no haber sentido ningún síntoma ni haber experimentado alteraciones en su vida cotidiana durante la transición a la menopausia. (*Code refers to participants who report they did not feel any symptoms or experience disruptions to their daily life during the menopause transition.*)	
Esto ya no es normal (*This is not normal anymore*)	Percepción de lo que es normal o no normal en la menopausia (sintomas o experencia). (*Perception of what is normal or not normal in menopause (symptoms or experience.*))	
El naturópata (*Naturopath*)	Cuando se refieren a tratamientos alternativos o "naturales". (*When referring to alternative or “natural” treatments.*)	**Menopause self-management and treatment options**
Hormona naturales *(Natural hormone*)	Este código se refiere a los participantes que distinguen la terapia hormonal sustitutiva y la medicación de las hormonas «naturales» de sus opiniones sobre estas hormonas «naturales». *(This code refers to the participants distinguishing hormone replacement therapy and medication from "natural" hormones their views of these "natural" hormones.)*	
Dudas de hormonas (*Hormone doubts*)	Utilice este código cuando los participantes describan sus dudas sobre las hormonas. Están sopesando pros y contras.* (Use this code when participants describe their doubt about hormones. They are weighing pros and cons.)*	
No soy pro hormonas (*I am not pro hormones*)	Los participantes expresan su oposición a la terapia hormonal o la decriben como algo malo. (*When participants express a strong stance against menopause hormone therapy or describe them as bad.)*	
Hormonas buenísimas (*Good hormones)*	Cuando la participante describe lo positivo o bueno de las hormonas (*When the participant describes the positive or good things about hormones.*)	
Vitaminas y ejercicio recomendado (*Vitamins and recommended exercise*)	Recomendación del proveedor de salud—vitaminas y ejercicio. (*Health care provider recommendation—vitamins and exercise)*	
Conversar con amigas (*Talking to friends*)	Amigas cómo fuentes de información (*Friends as sources of information.*)	**Informacion es poder** **(*information is power*)**
Conversar con familiars (*Talking to family)*	Familiares cómo fuentes de información. (*Relatives as sources of menopause information.*)	
Internet (*Internet)*	El internet cómo fuentes de información. (*The internet as source of menopause information.*)	
Television (*Television*)	Programas de televisión cómo fuentes de información. (*Television as sources of menopause information.*)	
Un proveedor de salud (Health p*rovider)*	Un proveedor de salud cómo fuentes de información (*Health provider as a source of menopause information.*)	
Información es poder (*Information is power*)	Se usa este code cuando las participantes describen lo que significa recibir información sobre la menopausia—poder, conocimiento para tomar mejor acción. (*This code is used when participants describe what it means to receive information about menopause—power, knowledge to take better action.)*	
Feliz compartiendo informacion (*Happy sharing information*)	La participante está dispuesta, disponible, y contenta a compartir información sobre la menopausia. (*Participant is willing, available, and happy to share information about menopause.*)	
Grupo de soporte (*Support group*)	Cuando participantes describen la necesidad de grupoos de soporte o de estar con personas que entienden la experiencia de menopausia—puede ser la idea de ellas crear un grupo o tener acceso a un grupo para no sentirse solas *(When participants describe the need for support groups or to be with people who understand the menopausal experience—it may be their idea to create a group or have access to a group so they do not feel alone.)*	
Poco conocimiento (*Little knowledge*)	Participantes describan su falta de conocimientos o sus escasos conocimientos sobre la menopausia. *(Participants describe their lack of knowledge or having little knowledge about menopause.)*	
Lo que deberia decir el proveedeor de salud (*What the health care provider should say*)	Este code se usa cuando las participantes hablan sobre sus expectativas para sus médicos tratantes—lo que deberían decir o hacer (*This code is used when participants talk about their expectations for their treating physicians—what they should say or do.)*	
No preparados para cambios (*Not ready for changes*)	El código se refiere a no estar preparado para los cambios de la mediana edad: síntomas, estado de ánimo, cambios en la vida. (*Code refers to not being ready for any midlife changes—could be symptoms, mood, life changes*.)	
Quiero conocer los síntomas (*I want to know the symptoms*)	Expectativas de conocimineto – síntomas (*Expectations of knowledge – symptoms*.)	
Quiero conocer la edad promedia (*I would like to know the average age*)	Expectativas de conocimineto—edad promedia (*Expectations of knowledge—average age*.)	
Conocer trastornos o problemas (*To know disorders or problems*)	Expectativas de conocimineto—los trastornos o problemas que puedan presentarse después y cómo tratarlos para prevenirlos. (*Expectations of knowledge—the disorders or problems that may occur later and how to treat them to prevent them.)*	
Conocer sobre las hormonas (*To know about hormones*)	Expectativas de conocimineto – hormonas (*Expectations of knowledge – hormones.)*	
Edades que deberias aprender de la menopausia (*Ages you should learn from menopause*)	Se usa cuando las participantes menciones las edades cuando se debe aprender sobre la menopausia. (*Used when participants mention the ages when to learn about menopause.)*	
Recibir informacion a edad joven (*Receiving information at a young age*)	Cuando las participantes hablan sobre la necesidad de preparar a las mujeres para la menopausia desde antes. (*When participants talk about the need to prepare women for menopause earlier in their lives, it is important to be prepared for menopause.*)	
No he probado nada (*I have not tested anything*)	Cuando los participantes expresan que no han utilizado ningún medicamento, tratamiento o estrategia de gestión para los síntomas de la menopausia. *(When participants express they have not used any medications, treatment, or management strategies for menopause symptoms.)*	

### Menopause is a stage of life (Una etapa de vida)

Focus group participants viewed menopause as a stage of life. This theme included descriptions of menopause as a biological process characterized by amenorrhea, loss of estrogen, and infertility. For example, one participant stated that menopause is *“when you stop having estrogen in your body and you no longer have eggs and you no longer have periods.*” Focus group data revealed this stage of life could be positive or negative depending on the individual situation. One participant described, *“for me it was a relief because I had a lot of problems. I had endometriosis, and mine [menopause] was a little bit more of a relief.”* Another positive aspect of this stage of life for women in relationships was no longer worrying about potential pregnancies: *“…for [my friend] it was the best news in the world. She was super happy, because she was single and if she had a relationship, there was no problem of getting pregnant.”* Yet, for other participants this stage of life was viewed negatively because it signaled a decline in health. One participant stated, *“with my 48 years, I'm starting to go down that curve. And for me, it means that I am going downhill.”* As a part of “going downhill” participants noted health status changes in weight, fatigue, mood, and blood pressure.

This theme also includes the viewpoint that menopause is a normal and natural stage. As one participant described, “*I took it as a normal process and never consulted [a doctor]*.” However, even among these participants, questions arose about what is considered “normal.” Participants consulted one another during focus group sessions to compare their symptoms and determine if others had similar experiences.

### Not wanting to become an old lady (No quererme hacer viejita)

This theme emerged from data describing menopause as a sign of aging. *"They think it's the worst thing that can happen to women, because we get older, because a lot of things happen to us. It's like ‘the time has come when we're not young anymore.’* While menopause itself was viewed as a normal stage of life with positive and negative aspects, aging was consistently described as an undesirable process. In addition, aging was described as a difficult reality to accept. Women perceived it to signal the end of beauty, health, and vitality. One participant stated:

*“I do not think it is neither ego nor arrogance, but the unwillingness to accept, an attitude of denying that it [aging] is going to happen to me, because it cannot happen to me because I have to keep myself always young and beautiful, slim, active and wonderful.”*

This negative view of aging and the connection between menopause and aging was further emphasized when participants described, *“the mockery…the pity, because it's like, yes, like ‘she's already old”* from friends, family, and general society. Participants emphasized that the negative view of society about aging impacts their self-perception during the MT.

### In our culture, we do not ask questions [about menopause]

Focus group participants described their reluctance to ask family members or healthcare providers about menopause. One woman recalled that she attempted to ask a friend but, “*I have not inquired any further because I feel kind of embarrassed, I don't know, kind of ashamed. I don't have that confidence with her to ask her anything.”* Some women attributed the hesitancy to talk about menopause to their culture. They felt that Latinas are reserved and quiet, “*in our culture, in our countries, we have a different way of being, let's put it this way, of opening ourselves to certain topics.”* Yet, participants also noted that in the U.S., they feel more open to having discussions about menopause. One participant described this by stating:

*“My grandmother was very reserved about everything. My mom the same way. Now I am the one who is starting to ask questions and do things. Maybe because we come to this country and in one's own country people are more quiet, more reserved, they don't ask questions, only if they tell you.”*

Of course, participants also noted that Hispanic/Latina women are not a homogenous group. Different cultures and nationalities of Hispanic/Latina women may be more open to discussions about menopause. A focus group participant from Spain explained, “*in Spain we are very open to talk about this kind of thing, and in my group of friends I am the last survivor [laughs], all of them have already gone through menopause.”* These differences may also impact relationships and the symptom experience. Participants like the one from Spain who were able to discuss menopause with a friend or family member felt some relief at knowing what to expect during the MT and received advice about how to manage symptoms.

### Family dynamics

A prominent theme across the focus groups was the impact of menopause on their family relationships. Participants shared that their family members often called them “crazy” when they were experiencing hot flashes or mood changes. These instances made them question themselves and if there was something wrong with them. One participant summed this by saying:

*“And then, of course, everybody's normal and you feel like you're crazy or different, because people will look at you like, ‘What's wrong with her? She seems like she’s so hot and there's no heat, the air conditioner is on.”*

Additionally, women emphasized that family members did not understand or were not empathetic to the changes they were undergoing during the MT. They suggested including family members in educational programs or healthcare visits about menopause to enhance their awareness. As one participant put it:

*“I think it would be very important for the family to know a little more about the subject, especially in relation to the symptoms, so that in that way, suddenly, the family members feel a little more empathy with the person who is going through the process. The symptoms are not going to be the same for everyone, but it would be good for them to know a little bit more about the symptoms.”*

Other participants acknowledged that their partners and children were understanding, supportive, and patient. However, these women felt guilty about how their menopause experience inconvenienced the family. Fatigue, mood changes, depressive symptoms, and anxiety were often referred to as being the most disruptive to the family. This is exemplified in the quotes below:

Participant 1: *“I'm always the one causing the problem [laughs]. My husband is a bread [from God], I mean, he's a good person, he doesn't go around mortifying me. If he sees that I'm upset and I want to pick a fight, he prefers to hit the road and leave me talking to myself.”*

Participant 2: *“My children already know that when I explode without any apparent reason it is not because I want to explode out of nowhere, it is because it is part of this biochemical and hormonal process.”*

Participants who were premenopausal and those who had not reported any symptoms during the MT expressed concerns about how symptoms would affect their relationships. The most common concern was the potential impact on their sex life. One participant stated:

“*My husband is younger than I am, and it is something that worries me, in terms of the sexual relations. So, it's something that I also share with him, and he also very much bears it in mind.”*

### Each body is different (Todo cuerpo es diferente)

Women typically emphasized that the menopause experience and its symptoms varied from person to person. Commonly discussed symptoms included joint pain, sleep disturbances, hot flashes, weight gain, mood changes, decreased libido, and hair loss. One participant stated:

*“It's a thing that is so changeable or so different from one person to another, that you can't predict... this will happen to the one with black hair, this will happen to the one with short hair.”*

Women also discussed how the severity of menopause symptoms may vary, with some women having severe symptoms and others experiencing none:

*“There are people that I have known, that it [menopause] gets them very hard, that is to say, that they have very serious symptoms, that they are bedridden, or they feel very bad, sick, you can say... I don't know, each organism is different, each person is different.”*

While women understood that symptoms may differ from person to person, the variability in symptoms and duration was a point of confusion. One participant stated:

*“Apart from the hot flashes, which I still have… I don't know why. It's been about four years since I entered menopause. But I still feel very hot, there are times when I wake up sweating. And another thing, that I started having pains in my breasts, I had to go to the doctor several times because my breasts hurt in specific points.”*

Women seemed relieved to hear that there were others in the group experiencing similar symptoms.; however, multiple women reported that they did not experience any of these symptoms. Participants expected that there would be a range of experiences and were interested in learning about each of these so that they could better prepare for their MT or can provide information to others.

### Menopause self-management and treatment options

Women reported multiple strategies for managing their menopause symptoms including lifestyle changes (i.e., diet, physical activity), herbal remedies (i.e., black cohosh, maca), melatonin, multivitamins, menopause hormone therapy, and bioidentical hormones. Participants emphasized the use of “natural” products for menopause symptoms:

*“I still struggle to find a natural lubricant to help me with the dryness. I was never able to find anything, because a lot of those things are expensive… But I did find a soap, like a gel, that was sold by some companies like Jafra, Avon or something, and each one is different, I don't have the name right now, but I did find a friend who sells some natural things, and that soap helps a lot.”*

Women learned about these naturopathic options from the internet, television programs, other women, and health providers. One participant stated:

*“I watched a television program…two women were interviewed… and they say that they didn't opt for hormones, but they opted for eating healthy, walking more and all that. And I've heard that there are natural teas that help you with that.”*

Most participants agreed that they preferred a healthy lifestyle change to any medications or hormonal options. In addition to diet and exercise, mental well-being and a positive mindset were viewed as important factors to managing menopause symptoms:

*“I will tell you again, how you take care of yourself is with exercise, with what you eat and that's it. What if you cut down on fats, sugar… a lot of water and exercise. That's what [I] always say to do at this age. If you are aware and you do it, believe me you will be able to overcome all those things they say you feel in menopause… I also think that it is more about psychological management than thinking about medication. I am not one of those people who takes medications, no. I don't like chemicals.”*

During the discussion, some women recommended the use of hormonal supplements, while others were prescribed bioidentical hormone pellets. When describing these treatments, women perceived them to be more natural because they were plant-based, and therefore safer:

*“I have a friend who recently had surgery for breast cancer, and she could not be treated with hormones, with synthetic hormones, because of the side effects. She had to have her womb removed. So, if there are natural hormones that don't have any side effects, obviously, I'm totally open to that, but whatever is natural.”*

Few participants used menopause hormone therapy and described their own experiences using them as positive. One participant stated, “*they helped me get on with my life – I have a lot of energy, I feel better.”* These women also emphasized that everyone may react differently.

Finally, this theme included descriptions from women who had not tried anything for their menopause symptoms. *"I haven't tried anything, and I would like to know what I can take or what I could do to help me because, to tell you the truth, I haven't tried anything.”* These participants either were not experiencing symptoms or did not know where to start and were seeking suggestions from the group.

### Information is power (información es poder)

One reason women participated in the focus group was to gain information about menopause that they could apply in their own lives and share with others. One woman put it simply as, *“information is power*.” When asked about the kind of information women should have about menopause, all focus groups responded similarly: what is menopause; signs and symptoms; symptom management strategies; communicating with family; and questions to ask health care providers. In addition, women agreed that receiving information earlier is better – beginning at least at age 35 years, but while in school is even better so that children can support their mothers:

*“I think it could start from the schools, like if they train the young people in that part so that they can understand their mothers a little bit, and I don't know how it could be with the husbands [laughs]. But, at least, with the youngsters for when they can understand their mothers in that sense.”*

Participants suggested sharing information in various forms, but the least favorable were brochures and handouts. Women emphasized the need for support groups to increase awareness and knowledge about menopause:

“*So, these groups are the ones that also help us to spread the word, because then, you see, it is you who also finds out through someone else. And that is how we are building a network of support for these very important issues.”*

Another suggestion was the use of community health workers. One participant shared that if trained in menopause health, she and her group of community health workers would educate community members and reach a wider audience.

## Discussion

The findings in this study support the need for culturally tailored menopause health interventions to improve menopause-related knowledge and symptom management. Nearly 60% of our focus group participants reported having little knowledge about menopause, which is consistent with prior research among non-Latina women [21]. Joint pain, sleep disturbances, fatigue, vasomotor symptoms, and weight gain/bloated were among the most bothersome symptoms women reported. Similarly, joint pain and fatigue were two of the most frequently reported symptoms among midlife Latinas in a cross-sectional online survey [7]. However, few studies have evaluated how bothersome Latinas view menopausal symptoms, which can impact quality of life and symptom management strategies. This qualitative study addressed these gaps using focus groups to better understand the experiences of menopause, including symptoms and management strategies among Latinas.

The first theme, “menopause as a stage of life (*una etapa de vida*)” is consistent with previous studies among midlife Latinas, which described menopause as *el cambio de vida* (the change of life) [10, 22]. In these earlier reports, the change of life referred to menopause as a new and normal stage in life with physical and psychological changes. While our focus group participants described menopause similarly, it is important to note that they also questioned what was considered normal during this life stage. In the descriptive survey, the majority of participants rated menopause positively, but during the focus group discussions there was no consensus about menopause being a positive or negative change. Our survey finding that the majority of women viewed menopause positively is consistent with earlier reports that Latinas have positive attitudes toward menopause [4, 10, 19]. In focus groups, women who viewed menopause positively stated that not having a period was a relief because they no longer had to worry about pregnancy, dysmenorrhea, or endometriosis-associated pain. Women who expressed more negative attitudes towards menopause associated menopause with aging and declining health. This finding is more consistent with prior work in New York with a mostly Puerto Rican group of Latinas who reported that menopause signaled a sudden decline in health [20]. Attitudes towards menopause may be related to sociocultural factors such as nationality and acculturation [21], which may explain the consistency of our findings with studies that have mainly focused on Mexican American women.

While women described positive and negative aspects of menopause, aging was viewed negatively across focus groups. This thematic finding differs from the Study of Women’s Health Across the Nation (SWAN), which reported that women’s attitudes about aging were generally positive; [22] however, Hispanic/Latina women had a more negative view of aging than African American and Black women in SWAN. Among Mexican women in Mexico City, only 7% had a negative self-perception of aging [23]. Our focus group participants stressed how society’s view of aging impacts their self-perception of aging. It is possible that our findings somewhat differ from prior studies because existing scales about attitudes toward aging do not consider participant’s interpretation of societal perceptions and expectations of older adults [24, 25].

Being silent and not asking questions about menopause is a common theme in other qualitative studies [4, 10, 22]. Multiple cultural perspectives have reservations surrounding open discussion of reproductive health, ranging from menarche to menopause [21]. Studies among Hispanic/Latina women report that these transitions are not just biological milestones but are imbedded with cultural and social significance, with some cultures offering additional familial support during these life stages [4]. However, a unique aspect of this theme in our research was a potential cultural paradigm shift among women with longer duration in the U.S and those with intergenerational communication. While still feeling reserved about discussing menopause, focus group participants felt they were more open to discussing the topic than their mothers or grandmothers. Similarly, participants felt that longer duration in the U.S. makes them more open to discussions about reproductive health. These findings are consistent with research demonstrating that greater acculturation is associated with a stronger sense of reproductive agency among Latinas [26, 27]. Additionally, recognizing that Latinas are a heterogenous group remains important in considering the context of this thematic understanding of openness surrounding conversations about menopause.

Research studies have shown that menopause symptoms may interfere with romantic and familial relationships [24–26]. Our findings add to this literature by highlighting that although participants feel misunderstood by family members during the MT, they also fear being a burden on them. This is consistent with evidence among non-Latino cultures as well [28]. However, the cultural value of *familismo* – the centrality of family in daily life [29] – may heighten feelings of distress for Hispanic/Latina women during the MT when their symptoms impact their ability to perform their usual roles in the family [30]. This was especially evident among participants who discussed the support they receive from family members and how critical that is to their menopause experience. This finding is like those reported by Im et al. (2009) [4], which underscored that although Hispanic/Latina women may rarely share their menopause symptom experience with family members, family support is essential during the transition. Another concern participants expressed during the MT is the impact of symptoms on their intimate relationships and how to approach such discussions; importantly, this theme has also emerged among non-Latina women who are perimenopausal [11, 28]. Thus, as focus group participants suggest, the involvement of intimate partners and family members is crucial in menopause interventions and health care.

The next two themes that emerged from our focus group data dealt with menopause symptoms and their management. These themes emphasize that because every person is different (*todo cuerpo es diferente*), they will experience different menopause symptoms and react to treatment differently. Many participants indicated that cultural values such as *simpatia* (sympathy) drove their symptom management, including hesitancy to confide symptoms in others who may perceive their struggles as burdensome. Despite this, participants found validation and empathy among their peers (*personalismo*) who shared their experiences. Specifically, participants relied on anecdotal experiences from one another including homeopathic remedies, for potential symptom management. Several participants indicated reservations about menopause hormone therapy and opted for self-management alternatives tested by friends and personality figures from popular television networks. There was also confusion around the different forms of menopause hormone therapy and what constituted “natural” versus synthetic.

The final theme of the analysis, *información es poder*, emphasizes that with increased knowledge, women can be better prepared for the changes associated with the MT and take action to minimize the impact of symptoms in their daily lives and relationships. The participants in our study indicated having little knowledge about menopause, which reflects larger trends surrounding the availability of menopause-related educational materials [17], particularly in Spanish. Women suggest earlier education about menopause in schools, which was similarly suggested by non-Latina participants in an online survey [11]. Women further suggested including partners and family members in menopause health education programs. We are aware of a single study evaluating menopause education and understanding among male partners of perimenopausal women [31]. Curiously, a lack of knowledge surrounding menopause hormone therapy may be related to the negative views participants shared about treatment [32]. Some participants stated they were more open to menopause hormone therapy if they had more information. As such, clinicians should also better educate themselves regarding the utility, potential side effects, and treatment goals surrounding menopause hormone therapy.

These themes align with previous research among African Americans and Asian Americans living in the U.S; [7, 22] for example, hesitancy surrounding menopause hormone therapy is a common concern due to institutional mistrust and healthcare accessibility [27, 33]. Additionally, previous studies have found a higher prevalence of family obligatory stress among patriarchal immigrant communities [28, 29], which may be a shared theme with our focus group. This is consistent with a prior qualitative study that reported the importance of family support to manage menopause symptoms [22]. Furthermore, there is a need to further investigate the nuances of attitudes towards menopause in this population, as previous studies found that attitudes towards menopause are correlated with perceived stress and menopause symptoms intensity [30].

There are several limitations and strengths to consider in this study. A potential limitation is that most (64%) of participants were Mexican, which can limit the transferability of findings to midlife Hispanic/Latina women from other geographic settings and nationalities. Additionally, focus groups were conducted using videoconferencing, which may have excluded women without an internet-enabled device. However, conducting focus groups online also allowed us to include women with transportation issues. A key strength of this study was the culturally diverse research staff which reflected the participant population [34]. As such, the focus group discussion and analyses were conducted in Spanish, which allowed us to accurately interpret and report findings in the participants’ own words.

## Conclusion

This study is one of the few to assess knowledge, attitudes, and experiences of the MT among midlife Latinas. We found that although Latinas report having a positive view of menopause, over half have little or no knowledge about the MT. Although considered a stage of life, Hispanic/Latina women are unsure of what to expect during the MT. In addition, women reported a negative attitude towards menopause hormone therapy and preferred “natural” alternative and complementary treatments to manage menopause symptoms. Findings suggest the need for culturally-tailored comprehensive menopause education earlier in life. The importance of involving family members in menopause education was also revealed. Findings support the paradigm shift that Hispanic/Latina women are engaging in more open dialogue about MT compared to previous generations, as evidenced by the discussions in the focus group and active participation in the focus group itself. Our next steps are to determine the best menopause messaging strategies and educational formats for midlife Latinas.

## Supplementary Information

Supplementary Material 1.

Supplementary Material 2.

## Data Availability

Data available on request from the authors. Contact the corresponding author at yamnia-cortes@uiowa.edu.

## References

[1] Bureau. UC. The Hispanic Population in the United States: 2019. Accessed February 7, 2022, https://www.census.gov/data/tables/2019/demo/hispanic-origin/2019-cps.html

[2] Moslimani MaN-B, L. Facts on Latinos in the U.S. Pew Research Center. Accessed September 9, 2023, https://www.pewresearch.org/hispanic/fact-sheet/latinos-in-the-us-fact-sheet/

[3] Gold EB, Sternfeld B, Kelsey JL, et al. Relation of demographic and lifestyle factors to symptoms in a multi-racial/ethnic population of women 40–55 years of age. Am J Epidemiol. 2000;152(5):463–73. 10981461 10.1093/aje/152.5.463

[4] Im EO, Lim HJ, Lee SH, Dormire S, Chee W, Kresta K. Menopausal symptom experience of Hispanic midlife women in the United States. Health Care Women Int. 2009;30(10):919–34. 10.1080/07399330902887582. 19742365 10.1080/07399330902887582 PMC2782818

[5] Green R, Polotsky AJ, Wildman RP, et al. Menopausal symptoms within a Hispanic cohort: SWAN, the Study of Women’s Health Across the Nation. Climacteric. 2010;13(4):376–84. 10.3109/13697130903528272. 20136411 10.3109/13697130903528272 PMC3268678

[6] Green R, Santoro NF, McGinn AP, et al. The relationship between psychosocial status, acculturation and country of origin in mid-life Hispanic women: data from the Study of Women’s Health Across the Nation (SWAN). Climacteric. 2010;13(6):534–43. 10.3109/13697131003592713. 20210631 10.3109/13697131003592713 PMC3677597

[7] Im EO. Ethnic differences in symptoms experienced during the menopausal transition. Health Care Women Int. 2009;30(4):339–55. 10.1080/07399330802695002. 19255887 10.1080/07399330802695002 PMC2670463

[8] Reeves AN, Lewis TT, Hood MM, et al. Does everyday discrimination account for the increased risk of vasomotor symptoms in Black women?: the Study of Women’s Health Across the Nation (SWAN). Menopause. 2024;31:484–93. 10.1097/GME.0000000000002357. 38595299 10.1097/GME.0000000000002357 PMC11126360

[9] Ayers B, Forshaw M, Hunter MS. The impact of attitudes towards the menopause on women’s symptom experience: a systematic review. Maturitas. 2010;65(1):28–36. 10.1016/j.maturitas.2009.10.016. 19954900 10.1016/j.maturitas.2009.10.016

[10] Villarruel AM, Harlow SD, Lopez M, Sowers M. El cambio de vida: conceptualizations of menopause and midlife among urban Latina women. Res Theory Nurs Pract. 2002;16(2):91–102. 10.1891/rtnp.16.2.91.53002. 12371435 10.1891/rtnp.16.2.91.53002

[11] Harper JC, Phillips S, Biswakarma R, et al. An online survey of perimenopausal women to determine their attitudes and knowledge of the menopause. Womens Health (Lond). 2022;18:17455057221106890. 10.1177/17455057221106890. 35758176 10.1177/17455057221106890 PMC9244939

[12] Kwak EK, Park HS, Kang NM. Menopause Knowledge, Attitude, Symptom and Management among Midlife Employed Women. J Menopausal Med. 2014;20(3):118–25. 10.6118/jmm.2014.20.3.118. 25580423 10.6118/jmm.2014.20.3.118 PMC4286656

[13] Haboush-Deloye A, Marquez E, Dunne R, Pharr JR. The Importance of Community Voice: Using Community-Based Participatory Research to Understand the Experiences of African American, Native American, and Latinx People During a Pandemic. Prev Chronic Dis. 2023;20:E12. 10.5888/pcd20.220152. 36893354 10.5888/pcd20.220152 PMC10038093

[14] Creswell JW, Creswell JD. Research design: Qualitative, quantitative, and mixed methods approaches. Sage publications; 2017.

[15] Health OoWs. A fact sheet from the office on women’s health: Top questions about menopause. Department of Health and Human Services. Updated 08/28/2017. Accessed 10/22/2024, https://owh-wh-d9-dev.s3.amazonaws.com/s3fs-public/documents/fact-sheet-menopause.pdf

[16] Sherman S. Defining the menopausal transition. *Am J Med*. Dec 19 2005;118 Suppl 12B:3–7. 10.1016/j.amjmed.2005.11.002 10.1016/j.amjmed.2005.11.002 16414321

[17] Harlow SD, Gass M, Hall JE, et al. Executive summary of the Stages of Reproductive Aging Workshop + 10: addressing the unfinished agenda of staging reproductive aging. J Clin Endocrinol Metab. 2012;97(4):1159–68. 10.1210/jc.2011-3362. 22344196 10.1210/jc.2011-3362 PMC3319184

[18] Saldana JM. The Coding Manual for Qualitative Researchers. 4th ed. London: SAGE Publications; 2021.

[19] Quintanilha M, Mayan MJ, Thompson J, Bell RC. Different approaches to cross-lingual focus groups: Lessons from a cross-cultural community-based participatory research project in the ENRICH Study. International Journal of Qualitative Methods. 2015:1–10. 10.1177/1609406915621419

[20] Younas A, Fàbregues S, Durante A, Ali P. Providing English and native language quotes in qualitative research: A call to action. Nurs Open. 2022;9(1):168–74. 10.1002/nop2.1115. 34725950 10.1002/nop2.1115 PMC8685880

[21] Tariq B, Phillips S, Biswakarma R, Talaulikar V, Harper JC. Women’s knowledge and attitudes to the menopause: a comparison of women over 40 who were in the perimenopause, post menopause and those not in the peri or post menopause. BMC Womens Health. 2023;23(1):460. 10.1186/s12905-023-02424-x. 37648988 10.1186/s12905-023-02424-x PMC10469514

[22] Im EO, Lee B, Chee W, Dormire S, Brown A. A national multiethnic online forum study on menopausal symptom experience. Nurs Res. 2010;59(1):26–33. 10.1097/NNR.0b013e3181c3bd69. 20010042 10.1097/NNR.0b013e3181c3bd69 PMC2882158

[23] Rivera-Ochoa FS, González-Herrera IV, Zacarías-Flores M, Correa-Muñoz E, Mendoza-Núñez VM, Sánchez-Rodríguez MA. Relationship between Self-Perception of Aging and Quality of Life in the Different Stages of Reproductive Aging in Mexican Women. Int J Environ Res Public Health. 2022;19(11):6839. 10.3390/ijerph19116839. 35682423 10.3390/ijerph19116839 PMC9180910

[24] Coslov N, Richardson MK, Woods NF. Symptom experience during the late reproductive stage and the menopausal transition: observations from the Women Living Better survey. Menopause. 2021;28(9):1012–25. 10.1097/GME.000000000000180511. 34313615 10.1097/GME.0000000000001805 PMC8549458

[25] Woods NF, Mitchell ES. Symptom interference with work and relationships during the menopausal transition and early postmenopause: observations from the Seattle Midlife Women’s Health Study. Menopause. 2011;18(6):654–61. 10.1097/gme.0b013e318205bd76. 21317821 10.1097/gme.0b013e318205bd76 PMC9668245

[26] Reame NK, Altemus M, Cortés Y, Jaime-Lara R. Perimenopause joint pain in urban Hispanic women: A qualitative study. Nurs Res. 2013;62(2):E1–118. 10.1097/NNR.0b013e318288c9c1.

[27] Doamekpor LA, Head SK, South E, et al. Determinants of Hormone Replacement Therapy Knowledge and Current Hormone Replacement Therapy Use. J Womens Health (Larchmt). 2023;32(3):283–92. 10.1089/jwh.2022.0342. 36459626 10.1089/jwh.2022.0342

[28] Im EO, Meleis AI. Women’s work and symptoms during midlife: Korean immigrant women. Women Health. 2001;33(1–2):83–103. 10.1300/J013v33n01_06. 11523642 10.1300/J013v33n01_06

[29] Zou P, Waliwitiya T, Luo Y, et al. Factors influencing healthy menopause among immigrant women: a scoping review. BMC Womens Health. 2021;21(1):189. 10.1186/s12905-021-01327-. 33957910 10.1186/s12905-021-01327-z PMC8101137

[30] Nosek M, Kennedy HP, Beyene Y, Taylor D, Gilliss C, Lee K. The effects of perceived stress and attitudes toward menopause and aging on symptoms of menopause. J Midwifery Womens Health Jul-Aug. 2010;55(4):328–34. 10.1016/j.jmwh.2009.09.005. 10.1016/j.jmwh.2009.09.005 PMC3661682 20630359

[31] Parish SJ, Faubion SS, Weinberg M, Bernick B, Mirkin S. The MATE survey: men’s perceptions and attitudes towards menopause and their role in partners’ menopausal transition. Menopause. 2019;26(10):1110–6. 10.1097/GME.0000000000001373. 31188286 10.1097/GME.0000000000001373 PMC6791510

[32] Barber K, Charles A. Barriers to accessing effective treatment and support for menopausal symptoms: a qualitative study capturing the behaviours, beliefs and experiences of key stakeholders. Patient Prefer Adherence. 2023;17:2971–80. 10.2147/PPA.S430203. 38027078 10.2147/PPA.S430203 PMC10657761

[33] Cuevas AG, O’Brien K. Racial centrality may be linked to mistrust in healthcare institutions for African Americans. J Health Psychol. 2019;24(14):2022–30. 10.1177/1359105317715092. 28810474 10.1177/1359105317715092

[34] Bazargan M, Cobb S, Assari S. Discrimination and medical mistrust in a racially and ethnically diverse sample of California adults. Ann Fam Med. 2021;19(1):4–15. 10.1370/afm.2632. 33431385 10.1370/afm.2632 PMC7800756

