# Supplementary material for: Información es poder (information is power): menopause knowledge, attitudes, and experiences in midlife Hispanic women and Latinas
Source: BMC Womens Health. 2024 Dec 2;24:633. doi: 10.1186/s12905-024-03434-z (PMC11613912; doi:10.1186/s12905-024-03434-z)
Supplement: Supplementary file 1 — Supplementary Material 1. [file 12905_2024_3434_MOESM1_ESM.docx]

Menopause Health Questionnaire

**SOCIODEMOGRAPHIC BACKGROUND**

| 1. Age:  2. Employment status: Unemployed Employed |
| --- |
| 3. Country of birth: |
| 4. Do you have health insurance? Yes No |

**MENSTRUAL PATTERNS AND SYMPTOMS**

5. How would you describe your current menstrual status?

Premenopause (before menopause; having regular periods)

Perimenopause/menopause transition (changes in periods, but have not gone 12 months in a row without a period)

Postmenopause (after menopause; answer 5a)

5a. Was your menopause:

Spontaneous (natural)

Surgical (removal of both ovaries)

Due to chemotherapy or radiation

Other (explain):

**6. Please indicate with a “X” how bothered you are now and in the past few weeks by any of the following:**

|  | **Not at all** | **A little bit** | **Quite a bit** | **Extremely** |
| --- | --- | --- | --- | --- |
| 1. I have hot flashes or night sweats |  |  |  |  |
| 1. I have difficulty getting to sleep or staying asleep |  |  |  |  |
| 1. I get heart palpitations or a sensation of butterflies in my chest or stomach |  |  |  |  |
| 1. I feel like my skin is crawling or itching |  |  |  |  |
| 1. I feel more tired than usual |  |  |  |  |
| 1. I have difficulty concentrating or forgetfulness |  |  |  |  |
| 1. I am more irritable than usual |  |  |  |  |
| 1. I feel more anxious than usual |  |  |  |  |
| 1. I have more depressed moods |  |  |  |  |
| 1. I am having mood swings |  |  |  |  |
| 1. I have crying spells |  |  |  |  |
| 1. I have headaches |  |  |  |  |
| 1. I need to urinate more often than usual |  |  |  |  |
| 1. I leak urine |  |  |  |  |
| 1. I have pain or burning when urinating |  |  |  |  |
| 1. I have bladder infections |  |  |  |  |
| 1. My vagina is dry |  |  |  |  |
| 1. I have vaginal itching |  |  |  |  |
| 1. I have an abnormal vaginal discharge |  |  |  |  |
| 1. I have vaginal infections |  |  |  |  |
| 1. I have pain during intercourse |  |  |  |  |
| 1. I have bleeding after intercourse |  |  |  |  |
| 1. I have decreased interest in sexual activity |  |  |  |  |
| 1. My stomach feel like it’s bloated or I’ve gained weight |  |  |  |  |
| 1. I have breast tenderness |  |  |  |  |
| 1. I have joint pains |  |  |  |  |

**7. How do you view menopause?**

**Positively.** For example, menopause means no more periods and no more worry about contraception. Menopause marks a new life phase.

**Negatively.** For example, menopause means a loss of fertility and loss of youth.

**Other:** ______________________________________________________________

**8. What are your current views regarding hormone therapy for menopause?**
❑ Positive. Hormone therapy is appropriate for some women.
❑ Negative. I don’t support the use of hormone therapy

**9.** How would you rate your knowledge about menopause?
❑ Very good ❑ Fair ❑ Moderately Good ❑ Little knowledge

10. How do you get your information about menopause? (Mark all that apply.)
❑ Books ❑ Internet ❑ Magazines

❑ Friends ❑ TV ❑ Healthcare provider
