# Supplementary material for: Información es poder (information is power): menopause knowledge, attitudes, and experiences in midlife Hispanic women and Latinas
Source: BMC Womens Health. 2024 Dec 2;24:633. doi: 10.1186/s12905-024-03434-z (PMC11613912; doi:10.1186/s12905-024-03434-z)
Supplement: Supplementary file 2 — Supplementary Material 2. [file 12905_2024_3434_MOESM2_ESM.docx]

# **Menopause Focus Group Question Guide**

***Thank you for joining us today. As you may know, the purpose of this focus group is for us to better understand what you know about, have heard, or experienced about menopause. We are also interested in your opinions about what should be included in a menopause education kit. During this session, we would like to hear from everyone. Please feel free to share your point of view even if it differs from what others have said. To protect your privacy, we would like for you to use your first name only. We will not use any names in our reports. We will audio record the session because we don't want to miss any of your comments, but we are not video recording. People often say very helpful things in these discussions and we can't write fast enough to get them all down. It is also important to respect each other’s privacy and not reveal any personal information about one another outside of this group. I have a few questions to guide our conversation. We also have a research assistant who will be helping me and taking notes about important topics that come up. Are there any questions before we start?***

1. **Introductions:**
   1. I would like you to introduce yourself.
   2. Tell us how you heard about the study and what made you decide to participate.
2. **Menopause Knowledge and Attitudes:**
   1. What does “menopause” mean to you?
   2. What are some assumptions or expectations?
   3. Where have you gotten this information about menopause?
   4. How have you discussed menopause with a health care provider?
   5. How have you discussed menopause with family members?
3. **Menopause Experience:**
   1. Tell me about any physical changes, or symptoms you have experienced that you think might be due to menopause. How has this affected your daily life or relationships?
   2. What sorts of things have you tried for your symptoms? How did you hear about these?
   3. Are there things that you’ve heard of other people trying for any of these issues? If so, what?
   4. We are also interested in hearing your thoughts about treatments like hormones for menopause symptoms. What have you heard and what are your thoughts?
   5. Are there any other health changes you have experienced?
4. **Menopause Education**
   1. As a woman approaches menopause, what information should she have?
   2. Where should she get this information and in what format (e.g., brochure, video, class, novela).
   3. Is there specific information that Latinas should have that might be different from other women?
   4. What might be important information for family members to understand about menopause?
   5. How likely are you to share information about menopause with family and friends?
   6. At what age should we begin to talk about menopause? What is information you wish you knew earlier?
   7. How might earlier information about menopause improve or worsen your experience?

***That concludes today’s conversation. Thank you for your time. Now, we will provide you with a short information session about menopause to hopefully answer some of the remaining questions you have about this important transition in a woman’s life.***
